# Methods to account for measured and unmeasured confounders in influenza relative vaccine effectiveness studies: A brief review of the literature

**DOI:** 10.1111/irv.12999

**Published:** 2022-05-11

**Authors:** Matthew M. Loiacono, Robertus Van Aalst, Darya Pokutnaya, Salaheddin M. Mahmud, Joshua Nealon

**Affiliations:** ^1^ Global Medical Evidence Generation Sanofi Swiftwater Pennsylvania USA; ^2^ Department of Modeling, Epidemiology, and Data Science Sanofi Lyon France; ^3^ Department of Health Sciences, University Medical Center Groningen University of Groningen Groningen The Netherlands; ^4^ Department of Health Services, Policy & Practice Brown University School of Public Health Providence Rhode Island USA; ^5^ Graduate School of Public Health University of Pittsburgh Pittsburgh Pennsylvania USA; ^6^ Vaccine and Drug Evaluation Centre, Department of Community Health Sciences University of Manitoba Winnipeg Canada; ^7^ School of Public Health, Li Ka Shing Faculty of Medicine The University of Hong Kong Hong Kong China; ^8^ Global Medical Evidence Generation Sanofi Lyon France

**Keywords:** comparative effectiveness research, confounding factors, epidemiologic, influenza vaccines, retrospective studies, review literature as topic

## Abstract

Observational seasonal influenza relative vaccine effectiveness (rVE) studies employ a variety of statistical methods to account for confounding and biases. To better understand the range of methods employed and implications for policy, we conducted a brief literature review. Across 37 included rVE studies, 10 different types of statistical methods were identified, and only eight studies reported methods to detect residual confounding, highlighting the heterogeneous state of the literature. To improve the comparability and credibility of future rVE research, researchers should clearly explain methods and design choices and implement methods to detect and quantify residual confounding.

Several differentiated seasonal influenza vaccines (SIVs) are authorized for use, promising enhanced protection over traditional egg‐based standard‐dose SIVs, which have been in use since the 1950s.^1^, ^2^ Relative vaccine efficacy/effectiveness (hereafter “rVE”) studies measure the *additional* protection conferred relative to other SIVs and must therefore detect smaller treatment effect sizes than absolute vaccine effectiveness studies that employ an unvaccinated or placebo reference group. Consequently, they require large sample sizes and are expensive to conduct using prospective designs, and randomized controlled trials (RCTs) to measure the relative performance of these vaccines have been conducted infrequently.

Instead, the rVE of SIVs has more frequently been assessed using observational study designs that estimate vaccine performance under “real‐world” conditions. Compared with RCTs, these studies are highly susceptible to specific sources of bias (e.g., the “healthy vaccinee effect”) and confounding (both measured and unmeasured), which can be substantial and have been shown to account for the entire observed reduction in outcome rates in some settings.^3^ Observational rVE studies have typically been conducted retrospectively using preexisting data, which can introduce specific challenges, particularly with exposure and outcome measurement.^4^

Although statistical methods and certain study designs exist to attempt to account for these biases, the presence of residual bias and confounding and resulting unreliable effect estimates remain important challenges to the broader adoption of comparative effectiveness observational research findings for healthcare policy.^5^ This has given rise to an increasing range and complexity of statistical methods, as well as methods to detect residual confounding in the SIV rVE literature.^6^ However, the rationale or justifications for choosing one method over another—a decision that can impact study results—are not always provided. To better understand the current state of the literature, the range of statistical methods employed, methods used to detect residual confounding, and the implications for vaccination policy, we conducted a review of the observational SIV rVE literature.

Using PubMed and Embase databases, we identified studies published between January 1, 2005, to July 1, 2021 and summarized their characteristics, including study design type, country of conduct, age of participants, statistical methods to account for confounding, and methods to detect residual confounding. We categorized statistical methods to account for confounding: individual matching, instrumental variable analysis, multivariable regression, prior event rate ratio (PERR) analysis, propensity score (PS)‐covariate adjustment, PS‐double robust estimation, PS‐inverse probability of treatment weighting (IPTW), PS‐matching, PS‐stratification, and PS‐weighted regression (herein referred to as weights). We categorized methods to detect residual confounding: negative control (an outcome unexpected to be affected by the exposure of interest, such that evidence of a treatment effect on this outcome indicates an underlying difference between treatment groups) or off‐season outcomes (estimating rVE outside of the influenza season, when a vaccine effect should be absent). Further details regarding the conduct of this review (Supporting Information), study inclusion (Figure S1), and study characteristics (Table S1) are available in the Appendix.

We identified 37 studies, 28 (75.7%) of which employed a cohort design and nine (24.3%) a case–control design (Table 1). The majority of studies were conducted in the United States (*n* = 29; 78.4%) and included adults aged 65 + years (*n* = 26; 70.3%). Among case–control studies, multivariable regression was the most commonly used method (*n* = 8; 89%), with the remainder using either individual matching or PS‐based methods (Figure 1). Among cohort studies, multivariable regression (*n* = 10; 36%) and IPTW were most common (*n* = 10; 36%).

**TABLE 1:** Characteristics of identified studies (*N* = 37)

**Study design**	
Cohort	28 (75.7%)
Case–control	9 (24.3%)
**Country of conduct**	
Canada	2 (5.4%)
Italy	3 (8.1%)
Multi‐country	1 (2.7%)
Spain	2 (5.4%)
United States	29 (78.4%)
**Age of study participants**	
6+ months	1 (2.7%)
4+ years	3 (8.1%)
18+ years	1 (2.7%)
2–17 years	3 (8.1%)
4–64 years	2 (5.4%)
17–49 years	1 (2.7%)
65+ years	26 (70.3%)

**FIGURE 1:**
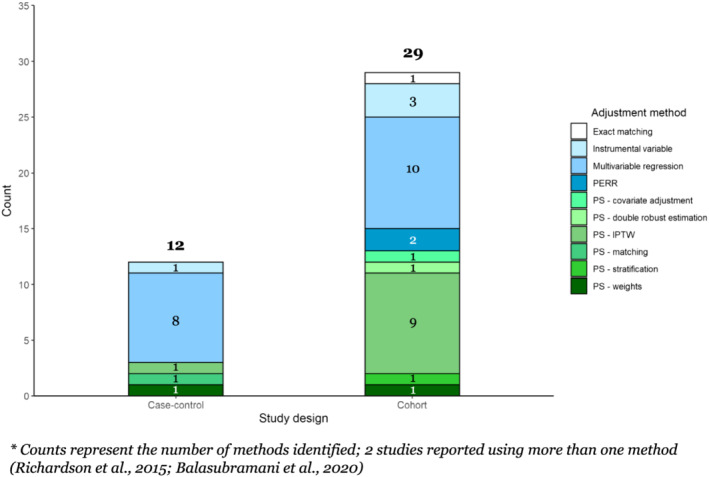
Count of statistical methods used*, by study design

The majority of studies were published between 2019 and 2021, with three or fewer studies published annually from 2009 to 2018 (Figure 2). From 2009 to 2017, multivariable regression was most often used. From 2019 onward, the use of IPTW increased and became predominant, and other methods were introduced, including instrumental variable and PERR analyses. Methods to detect residual confounding were reported in only eight studies (22%) (Figure 3). Off‐season outcomes were used sporadically, with negative control outcomes reportedly being used more frequently—once in 2019, twice in 2020, and in three out of the six studies published in 2021.

**FIGURE 2:**
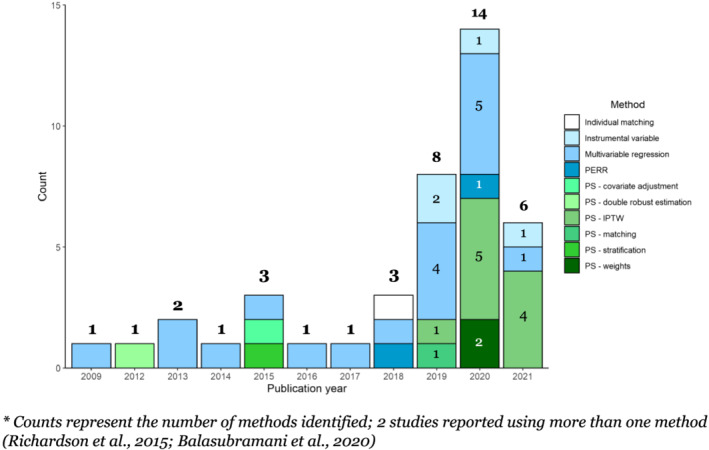
Count of statistical methods used*, by publication year

**FIGURE 3:**
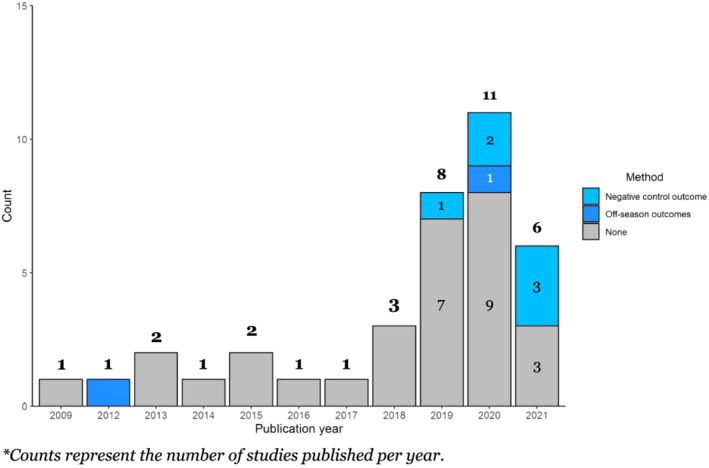
Count of studies assessing residual confounding (specific method used)*, by publication year

These findings underscore the heterogeneous state of the observational SIV rVE literature and the wide range of statistical methods employed over the past 15 years, aligned with conclusions of a recent systematic review of rVE estimation methods.^6^ Most studies adjusted for measured confounding by including a large number of classical potential confounders in multivariable models. However, fewer than 30% of studies accounted for unmeasured confounders and/or implemented methods to detect residual confounding, implying a degree of inaccuracy in estimated effect sizes. Unbiased treatment effects cannot typically be generated without randomization, and unmeasured confounding represents a clear source of inaccuracy that could have been addressed through primary or sensitivity analyses, improving robustness. Methods identified in our review included (1) those used to *qualitatively assess* the potential impact of unmeasured confounders on the observed treatment effect, including negative control outcomes or off‐season time periods, and (2) those attempting to *account for* unmeasured confounding, including PERR analysis [used in two studies], adjusting the observed treatment effect with the observed imbalance in outcomes during a baseline period (where no difference is expected); and instrumental variable analysis [used in four studies],—using a variable that has a causal effect on the exposure, and is associated with the outcome, but only affects the outcome indirectly through the exposure—to estimate the causal effect of vaccination.^7^

Additional options to detect or control for unmeasured confounding include the use of case‐only study designs that minimize some forms of bias and, depending on data availability, the application of additional sensitivity analyses, sub‐studies, and specialized adjustment methods.^8^ A relatively simple method that should be more widely employed is the “E‐value” approach, which estimates the minimum strength of association an unmeasured confounder must have with the treatment and outcome of interest as to invalidate reported findings.^9^ Consistent adoption and transparent reporting of such methods would increase observational study robustness and allow readers and policymakers to contextualize and operationalize observational study results with more confidence.

In addition to these explicit differences in design choice, individual study results are affected by implementation decisions made by study teams, including choice of data sources, covariate selection and modeling strategies, matching algorithms, and other statistical definitions. These choices are particularly impactful in modifying small overall effect sizes, rendering direct comparison of findings from different rVE studies challenging.^6^ For example, we identified studies conducted during the same influenza season, enrolling comparable populations (community‐dwelling older adults), employing similar methods (PS‐IPTW), and assessing similar outcomes, which generated directionally opposite results. Izurieta et al reported an rVE of 5.3% (95% CI: 3.3%–7.3%) for high‐dose versus adjuvanted SIV against influenza‐related hospital encounters during the 2017/2018 season,^10^ whereas Boikos et al reported an rVE of −7.7% (−12.8% to −2.3%) against influenza‐related medical encounters for the same vaccines and season.^11^ If study protocols are unavailable and the rationale behind key design decisions are unexplained, meaningful interpretation of these findings—which may have been influenced by conscious or unconscious biases of the investigators—becomes impossible.^12^ In another example of divergent technique, Balasubramani et al defined rVE as the relative improvement in absolute VE (VE1/VE2‐1), whereas rVE is normally defined as 1—relative risk/odds ratio between vaccine groups.^13^ This subtlety is likely to be missed by most readers, yet directly impacts the comparability of reported estimates.

Illustrating the potential impact of implementation decisions, Robison and Thomas explored the effect of matching factors on rVE, reporting a wide range of rVE estimates for high‐dose versus standard‐dose SIVs against laboratory‐confirmed influenza hospitalizations, spanning from as low as 3.4% (−6% to 29%) to as high as 30.7% (8% to 48%) in a fully adjusted model.^14^ Simulation studies have similarly sought to quantify the degree to which design decisions can bias effect estimates. Austin and Stuart previously demonstrated that decisions such as foregoing imposed caliper restrictions (for PS‐matching) or IPTW restrictions (e.g., excluding individuals with extreme weights) can bias estimates by 5% or more.^15^

There are several limitations to this review. Although we described the use of methods to account for confounding in observational rVE research, assessment of the quantitative impact of specific implementation decisions was outside of scope. Additionally, we categorized similar methods, some of which included more than one model type (e.g., logistic and Cox regressions categorized as multivariable regression), which may have concealed some further methodological heterogeneity.

Considering the inconsistency and complexity described in this review of rVE literature, it is unsurprising that many of these studies have generated low‐quality evidence, as assessed by independent public health organizations.^1^ To improve the comparability and credibility of future SIV observational rVE studies, researchers should clearly explain methods and design choices and consistently implement methods to detect and quantify residual confounding.

## ETHICS STATEMENT

As this was a retrospective review of the literature, no ethics approvals were required. As this was a retrospective review of the literature, patient consent was not applicable or required.

## AUTHOR CONTRIBUTIONS

**Matthew Loiacono:** Conceptualization; data curation; formal analysis; project administration; validation; visualization. **Rob Van Aalst:** Conceptualization; investigation; supervision; visualization. **Darya Pokutnaya:** Conceptualization; data curation; formal analysis; methodology; validation; visualization. **Salaheddin Mahmud:** Conceptualization; investigation; methodology; supervision; visualization. **Joshua Nealon:** Conceptualization; investigation; project administration; supervision; validation; visualization.

### PEER REVIEW

The peer review history for this article is available at https://publons.com/publon/10.1111/irv.12999.

## Supporting information




**Figure S1.** PRISMA flow diagram
**Table S1.** Characteristics of included studies (N = 37).

## Data Availability

Search queries used and all studies included in this review, including identified characteristics, are available in the Appendix.
