# Supplementary material for: Methods to account for measured and unmeasured confounders in influenza relative vaccine effectiveness studies: A brief review of the literature
Source: Influenza Other Respir Viruses. 2022 May 11;16(5):846–50. doi: 10.1111/irv.12999 (PMC9343322; doi:10.1111/irv.12999)
Supplement: Supplementary file 1 — Figure S1. PRISMA flow diagram Table S1. Characteristics of included studies (N = 37). [file IRV-16-846-s001.docx]

**Methods to account for measured and unmeasured confounders in influenza relative vaccine effectiveness studies: a brief review of the literature**

**Appendix**

**Supplemental Methods**

**Literature Review**

We conducted a systematic review of published literature using the PRISMA framework to identify observational studies assessing the relative vaccine effectiveness (rVE) of seasonal influenza vaccines (SIVs)^[[1]](#footnote-1)^. Using PubMed and Embase databases, we identified studies published from January 1, 2005 to July 1, 2021, limiting our search to studies conducted in humans, published in English, and where full-text articles were available. Papers eligible for inclusion were observational studies reporting the rVE of differentiated SIVs. Screening of all identified studies was performed independently by two co-authors (DP & MML); any discrepancies were re-assessed amongst the co-authors before coming to a consensus.

**Search Queries**

Final PubMed search:

((((((influenza, human[MeSH Terms]) OR (flu[Title/Abstract]) OR (influenza[Title/Abstract])) AND (vacci*[Title/Abstract])) AND ((observational[Title/Abstract]) OR (cross-sectional[Title/Abstract]) OR (case-control[Title/Abstract]) OR (cohort[Title/Abstract]) OR (retrospective[Title/Abstract]) OR (prospective[Title/Abstract]) OR (ecological[Title/Abstract]) OR (longitudinal[Title/Abstract)) AND ((comparative effectiveness research[MeSH Terms]) OR (research, comparative effectiveness[MeSH Terms]) OR ("relative effectiveness"[Title/Abstract]) OR (rVE[Title/Abstract]) OR (compari*[Title/Abstract]) OR ("comparative effectiveness"[Title/Abstract]))))) AND (("2005"[Date - Publication] : "3000"[Date - Publication]))) AND (English[Language])

Final Embase search:

(influenza:ab,ti OR flu:ab,ti) AND ('influenza vaccine':ab,ti OR 'influenza vaccination':ab,ti OR vacci*:ab,ti) AND ('observational':ab,ti OR 'cross sectional':ab,ti OR 'case control':ab,ti OR 'cohort':ab,ti OR retrospective:ab,ti OR prospective:ab,ti) AND (rve:ab,ti OR compari*:ab,ti OR 'relative vaccine effectiveness':ab,ti OR 'comparative effectiveness':ab,ti OR 'relative effectiveness':ab,ti) AND [2005-2021]/py AND [english]/lim

**Supplemental Figure 1. PRISMA flow diagram**

**
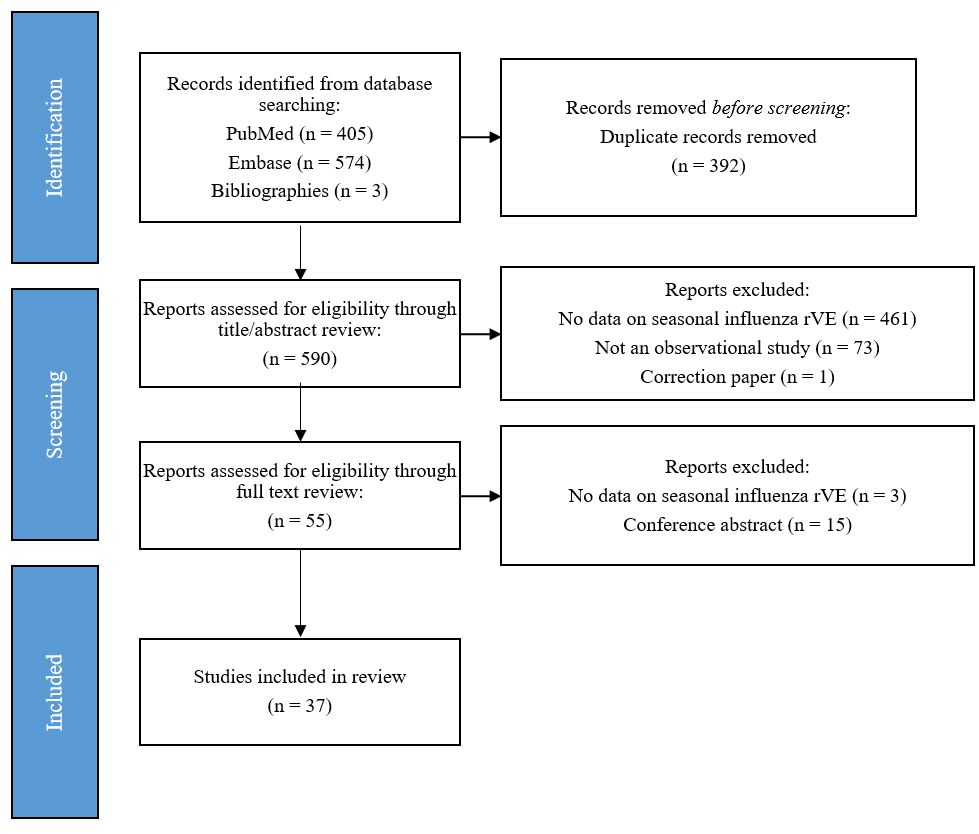
**

| Author, Year  **Supplemental Table 1. Characteristics of included studies (N=37).** | Study Design | Country | Age | Statistical adjustment Method(s) | Residual confounding assessment |
| --- | --- | --- | --- | --- | --- |
| Eick, 2009^1^ | Cohort | US | 17-49 yrs | Multivariable regression | None |
| Manninno, 2012^2^ | Cohort | Italy | 65+ yrs | PS - double robust estimation | Off-season outcomes |
| Van Buynder, 2013^3^ | Case-control | Canada | 65+ yrs | Multivariable regression | None |
| Puig-Barberà, 2013^4^ | Cohort | Spain | 65+ yrs | Multivariable regression | None |
| Puig-Barberà, 2014^5^ | Cohort | Spain | 65+ yrs | Multivariable regression | None |
| Richardson, 2015^6^ | Cohort | US | 65+ yrs | 1) PS – stratification  2) PS - covariate adjustment | None |
| Izurieta, 2015^7^ | Cohort | US | 65+ yrs | Multivariable regression | None |
| Chung, 2016^8^ | Case-control | US | 2-17 yrs | Multivariable regression | None |
| Shay, 2017^9^ | Cohort | US | 65+ yrs | Multivariable regression | None |
| Robison, 2018^10^ | Cohort | US | 65+ yrs | Exact matching | None |
| Buchan, 2018^11^ | Case-control | Canada | 2-17 yrs | Multivariable regression | None |
| Young-Xu, 2018^12^ | Cohort | US | 65+ yrs | PERR | None |
| Bruxvoort, 2019^13^ | Case-control | US | 4+ yrs | Multivariable regression | None |
| Lu, 2019^14^ | Cohort | US | 65+ yrs | Multivariable regression | None |
| Lapi, 2019^15^ | Case-control | Italy | 65+ yrs | PS - matching | None |
| Young-Xu, 2019^16^ | Cohort | US | 65+ yrs | Instrumental variable | Negative control outcome |
| Chung, 2019^17^ | Case-control | US | 2-17 yrs | Multivariable regression | None |
| Izurieta, 2019^18^ | Cohort | US | 65+ yrs | PS - IPTW | None |
| DeMarcus, 2019^19^ | Case-control | Multi | 6+ mos | Multivariable regression | None |
| van Aalst, 2019^20^ | Cohort | US | 65+ yrs | Instrumental variable | None |
| Izurieta, 2020^21^ | Cohort | US | 65+ yrs | PS - IPTW | Negative control outcome |
| Balasubramani, 2020^22^ | Case-control | US | 65+ yrs | 1) Multivariable regression  2) Instrumental variable  3) PS - weights  4) PS - IPTW | None |
| Cocchio, 2020^23^ | Cohort | Italy | 65+ yrs | Multivariable regression | None |
| Paudel, 2020^24^ | Cohort | US | 65+ yrs | Multivariable regression | None |
| Butler, 2020^25^ | Cohort | US | 18+ yrs | PS - weights | None |
| Boikos, 2020^26^ | Cohort | US | 4+ yrs | Multivariable regression | None |
| Izurieta, 2020^27^ | Cohort | US | 65+ yrs | PS - IPTW | None |
| van Aalst, 2020^28^ | Cohort | US | 65+ yrs | PERR | Negative control outcome |
| Pelton, 2020^29^ | Cohort | US | 65+ yrs | PS - IPTW | None |
| Divino, 2020^30^ | Cohort | US | 4-64 yrs | PS - IPTW | None |
| Young-Xu, 2020^31^ | Cohort | US | 65+ yrs | Multivariable regression | Off-season outcomes |
| Boikos, 2021^32^ | Cohort | US | 65+ yrs | PS - IPTW | None |
| Krishnarajah, 2021^33^ | Cohort | US | 4-64 yrs | PS - IPTW | Negative control outcome |
| Boikos, 2021^34^ | Cohort | US | 4+ yrs | PS - IPTW | None |
| van Aalst, 2021^35^ | Cohort | US | 65+ yrs | Instrumental variable | None |
| Doyle, 2021^36^ | Case-control | US | 65+ yrs | Multivariable regression | Negative control outcome |
| Pelton, 2021^37^ | Cohort | US | 65+ yrs | PS - IPTW | Negative control outcome |

**References**

1. Eick AA, Wang Z, Hughes H, Ford SM, Tobler SK. Comparison of the trivalent live attenuated vs. inactivated influenza vaccines among US military service members. *Vaccine.* 2009;27(27):3568-3575.

2. Mannino S, Villa M, Apolone G, et al. Effectiveness of adjuvanted influenza vaccination in elderly subjects in northern Italy. *American journal of epidemiology.* 2012;176(6):527-533.

3. Van Buynder P, Konrad S, Van Buynder J, et al. The comparative effectiveness of adjuvanted and unadjuvanted trivalent inactivated influenza vaccine (TIV) in the elderly. *Vaccine.* 2013;31(51):6122-6128.

4. Puig-Barberà J, Natividad-Sancho A, Calabuig-Pérez J, et al. MF59-adjuvanted and virosomal influenza vaccines for preventing influenza hospitalization in older people: comparative effectiveness using the Valencia health care information system. *Vaccine.* 2013;31(37):3995-4002.

5. Puig-Barberà J, Natividad-Sancho A, Calabuig-Pérez J, et al. Intradermal and virosomal influenza vaccines for preventing influenza hospitalization in the elderly during the 2011–2012 influenza season: A comparative effectiveness study using the Valencia health care information system. *Vaccine.* 2014;32(42):5447-5454.

6. Richardson DM, Medvedeva EL, Roberts CB, Linkin DR. Comparative effectiveness of high-dose versus standard-dose influenza vaccination in community-dwelling veterans. *Clinical infectious diseases.* 2015;61(2):171-176.

7. Izurieta HS, Thadani N, Shay DK, et al. Comparative effectiveness of high-dose versus standard-dose influenza vaccines in US residents aged 65 years and older from 2012 to 2013 using Medicare data: a retrospective cohort analysis. *The Lancet Infectious diseases.* 2015;15(3):293-300.

8. Chung JR, Flannery B, Thompson MG, et al. Seasonal effectiveness of live attenuated and inactivated influenza vaccine. *Pediatrics.* 2016;137(2).

9. Shay DK, Chillarige Y, Kelman J, et al. Comparative effectiveness of high-dose versus standard-dose influenza vaccines among US medicare beneficiaries in preventing postinfluenza deaths during 2012–2013 and 2013–2014. *The Journal of infectious diseases.* 2017;215(4):510-517.

10. Robison SG, Thomas AR. Assessing the effectiveness of high-dose influenza vaccine in preventing hospitalization among seniors, and observations on the limitations of effectiveness study design. *Vaccine.* 2018;36(45):6683-6687.

11. Buchan SA, Booth S, Scott AN, et al. Effectiveness of live attenuated vs inactivated influenza vaccines in children during the 2012-2013 through 2015-2016 influenza seasons in Alberta, Canada: a Canadian Immunization Research Network (CIRN) study. *JAMA pediatrics.* 2018;172(9):e181514-e181514.

12. Young-Xu Y, Van Aalst R, Mahmud SM, et al. Relative vaccine effectiveness of high-dose versus standard-dose influenza vaccines among Veterans Health Administration patients. *The Journal of infectious diseases.* 2018;217(11):1718-1727.

13. Bruxvoort KJ, Luo Y, Ackerson B, et al. Comparison of vaccine effectiveness against influenza hospitalization of cell-based and egg-based influenza vaccines, 2017–2018. *Vaccine.* 2019;37(39):5807-5811.

14. Lu Y, Chillarige Y, Izurieta HS, et al. Effect of age on relative effectiveness of high-dose versus standard-dose influenza vaccines among US Medicare beneficiaries aged≥ 65 years. *The Journal of infectious diseases.* 2019;220(9):1511-1520.

15. Lapi F, Marconi E, Simonetti M, et al. Adjuvanted versus nonadjuvanted influenza vaccines and risk of hospitalizations for pneumonia and cerebro/cardiovascular events in the elderly. *Expert review of vaccines.* 2019;18(6):663-670.

16. Young-Xu Y, Snider JT, van Aalst R, et al. Analysis of relative effectiveness of high-dose versus standard-dose influenza vaccines using an instrumental variable method. *Vaccine.* 2019;37(11):1484-1490.

17. Chung JR, Flannery B, Ambrose CS, et al. Live attenuated and inactivated influenza vaccine effectiveness. *Pediatrics.* 2019;143(2).

18. Izurieta HS, Chillarige Y, Kelman J, et al. Relative effectiveness of cell-cultured and egg-based influenza vaccines among elderly persons in the United States, 2017–2018. *The Journal of infectious diseases.* 2019;220(8):1255-1264.

19. DeMarcus L, Shoubaki L, Federinko S. Comparing influenza vaccine effectiveness between cell-derived and egg-derived vaccines, 2017–2018 influenza season. *Vaccine.* 2019;37(30):4015-4021.

20. van Aalst R, Russo EM, Neupane N, et al. Economic assessment of a high-dose versus a standard-dose influenza vaccine in the US Veteran population: Estimating the impact on hospitalization cost for cardio-respiratory disease. *Vaccine.* 2019;37(32):4499-4503.

21. Izurieta HS, Lu M, Kelman J, et al. Comparative effectiveness of influenza vaccines among US Medicare beneficiaries ages 65 years and older during the 2019-20 season. *Clinical Infectious Diseases: an Official Publication of the Infectious Diseases Society of America.* 2020.

22. Balasubramani G, Choi WS, Nowalk MP, et al. Relative effectiveness of high dose versus standard dose influenza vaccines in older adult outpatients over four seasons, 2015–16 to 2018–19. *Vaccine.* 2020;38(42):6562-6569.

23. Cocchio S, Gallo T, Del Zotto S, et al. Preventing the risk of hospitalization for respiratory complications of influenza among the elderly: is there a better influenza vaccination strategy? A retrospective population study. *Vaccines.* 2020;8(3):344.

24. Paudel M, Mahmud S, Buikema A, et al. Relative vaccine efficacy of high-dose versus standard-dose influenza vaccines in preventing probable influenza in a Medicare Fee-for-Service population. *Vaccine.* 2020;38(29):4548-4556.

25. Butler AM, Layton JB, Dharnidharka VR, et al. Comparative effectiveness of high-dose versus standard-dose influenza vaccine among patients receiving maintenance hemodialysis. *American Journal of Kidney Diseases.* 2020;75(1):72-83.

26. Boikos C, Sylvester GC, Sampalis JS, Mansi JA. Relative effectiveness of the cell-cultured quadrivalent influenza vaccine compared to standard, egg-derived quadrivalent influenza vaccines in preventing influenza-like illness in 2017–2018. *Clinical Infectious Diseases.* 2020;71(10):e665-e671.

27. Izurieta HS, Chillarige Y, Kelman J, et al. Relative effectiveness of influenza vaccines among the United States elderly, 2018–2019. *The Journal of infectious diseases.* 2020;222(2):278-287.

28. van Aalst R, Gravenstein S, Mor V, et al. Comparative effectiveness of high dose versus adjuvanted influenza vaccine: A retrospective cohort study. *Vaccine.* 2020;38(2):372-379.

29. Pelton SI, Divino V, Shah D, et al. Evaluating the Relative Vaccine Effectiveness of Adjuvanted Trivalent Influenza Vaccine Compared to High-Dose Trivalent and Other Egg-Based Influenza Vaccines among Older Adults in the US during the 2017–2018 Influenza Season. *Vaccines.* 2020;8(3):446.

30. Divino V, Krishnarajah G, Pelton SI, et al. A real-world study evaluating the relative vaccine effectiveness of a cell-based quadrivalent influenza vaccine compared to egg-based quadrivalent influenza vaccine in the US during the 2017–18 influenza season. *Vaccine.* 2020;38(40):6334-6343.

31. Young-Xu Y, Snider JT, Mahmud SM, et al. High-dose influenza vaccination and mortality among predominantly male, white, senior veterans, United States, 2012/13 to 2014/15. *Eurosurveillance.* 2020;25(19):1900401.

32. Boikos C, Fischer L, O’Brien D, Vasey J, Sylvester GC, Mansi JA. Relative Effectiveness of Adjuvanted Trivalent Inactivated Influenza Vaccine Versus Egg-derived Quadrivalent Inactivated Influenza Vaccines and High-dose Trivalent Influenza Vaccine in Preventing Influenza-related Medical Encounters in US Adults≥ 65 Years During the 2017–2018 and 2018–2019 Influenza Seasons. *Clinical Infectious Diseases.* 2021.

33. Krishnarajah G, Divino V, Postma MJ, et al. Clinical and Economic Outcomes Associated with Cell-Based Quadrivalent Influenza Vaccine vs. Standard-Dose Egg-Based Quadrivalent Influenza Vaccines during the 2018–19 Influenza Season in the United States. *Vaccines.* 2021;9(2):80.

34. Boikos C, Fischer L, O’Brien D, Vasey J, Sylvester GC, Mansi JA. Relative Effectiveness of the Cell-derived Inactivated Quadrivalent Influenza Vaccine Versus Egg-derived Inactivated Quadrivalent Influenza Vaccines in Preventing Influenza-related Medical Encounters During the 2018–2019 Influenza Season in the United States. *Clinical Infectious Diseases.* 2021.

35. van Aalst R, Russo EM, Neupane N, et al. Comparing the impact of high-dose versus standard dose influenza vaccines on hospitalization cost for cardiovascular and respiratory diseases: Economic assessment in the US Veteran population during 5 respiratory seasons using an instrumental variable method. *Vaccine.* 2021;39:A51-A55.

36. Doyle JD, Beacham L, Martin ET, et al. Relative and absolute effectiveness of high-dose and standard-dose influenza vaccine against influenza-related hospitalization among older adults—United States, 2015–2017. *Clinical Infectious Diseases.* 2021;72(6):995-1003.

37. Pelton SI, Divino V, Postma MJ, et al. A retrospective cohort study assessing relative effectiveness of adjuvanted versus high-dose trivalent influenza vaccines among older adults in the United States during the 2018–19 influenza season. *Vaccine.* 2021;39(17):2396-2407.

1. Page MJ, McKenzie JE, Bossuyt PM, et al. The PRISMA 2020 statement: an updated guideline for reporting systematic reviews. *Bmj.* 2021;372. [↑](#footnote-ref-1)
